# Supplementary material for: A Likelihood Approach for Real-Time Calibration of Stochastic Compartmental Epidemic Models
Source: PLoS Comput Biol. 2017 Jan 17;13(1):e1005257. doi: 10.1371/journal.pcbi.1005257 (PMC5240920; doi:10.1371/journal.pcbi.1005257)
Supplement: S1 File — (TAR.GZ) [file pcbi.1005257.s014.tar.gz › HSPH_Online-SI-Revision/MSS10/n10-mild/n10-mild_NEW2_summary2.pdf]

peak -8	3w spec	3w cum	5w spec	5w cum	end	3w spec LOG	3w cum LOG	5w spec LOG	5w cum LOG	end LOG
mean	233.4	186.6	154.	165.6	68.6	0.5	0.4	0.6	0.4	0.4
median	86.3	79.	78.4	78.8	63.3	0.5	0.3	0.5	0.4	0.4
quantile .1	29.5	23.7	45.	32.8	36.3	0.1	0.1	0.2	0.2	0.2
quantile .9	652.5	439.	342.9	388.4	103.	0.9	0.7	1.	0.2	0.8
36	39	29	35	18						

peak -4	3w spec	3w cum	5w spec	5w cum	end	3w spec LOG	3w cum LOG	5w spec LOG	5w cum LOG	end LOG
mean	61.1	60.4	53.1	51.2	51.	0.3	0.2	0.4	0.3	0.3
median	44.8	39.8	43.7	38.3	43.2	0.3	0.2	0.3	0.2	0.3
quantile .1	24.9	22.4	29.9	22.2	19.3	0.1	0.1	0.1	0.1	0.1
quantile .9	70.3	73.4	83.1	68.1	80.7	0.5	0.4	0.9	0.1	0.6
41	45	30	38	17						

peak	3w spec	3w cum	5w spec	5w cum	end	3w spec LOG	3w cum LOG	5w spec LOG	5w cum LOG	end LOG
mean	37.7	29.6	51.2	31.5	40.9	0.2	0.1	0.3	0.2	0.2
median	31.1	24.9	41.9	25.8	37.2	0.2	0.1	0.3	0.1	0.2
quantile .1	19.8	16.1	27.6	16.4	17.5	0.1	0.1	0.1	0.1	0.1
quantile .9	52.6	40.6	67.3	43.8	55.3	0.3	0.2	0.5	0.1	0.4
35	44	26	36	17						

peak +4	3w spec	3w cum	5w spec	5w cum	end	3w spec LOG	3w cum LOG	5w spec LOG	5w cum LOG	end LOG
mean	54.8	35.6	252.1	39.3	49.9	0.4	0.2	0.7	0.2	0.3
median	45.2	32.	62.	36.9	47.5	0.3	0.2	0.6	0.2	0.3
quantile .1	26.6	17.2	33.	17.7	18.8	0.1	0.1	0.2	0.1	0.1
quantile .9	65.5	46.2	97.3	50.5	68.3	0.6	0.3	1.	0.1	0.5
31	38	27	31	20						

peak +8	3w spec	3w cum	5w spec	5w cum	end	3w spec LOG	3w cum LOG	5w spec LOG	5w cum LOG	end LOG
mean	234.7	772.6	297.3	837.6	856.7	0.5	0.3	0.8	0.4	0.5
median	59.1	44.7	78.3	51.2	61.5	0.5	0.3	0.8	0.3	0.4
quantile .1	38.1	25.7	51.1	28.2	32.2	0.2	0.1	0.4	0.1	0.2
quantile .9	177.5	129.2	734.	152.2	155.8	0.9	0.4	1.3	0.1	0.7
31	32	32	30	27	{}	{}	{}	{}	{}	{}
